# Effect of Exposure Temperature on the Crashworthiness of Carbon/Epoxy Composite Rectangular Tubes Under Quasi-Static Compression

**DOI:** 10.3390/polym12092028

**Published:** 2020-09-05

**Authors:** Tamer A. Sebaey

**Affiliations:** 1 Engineering Management Department, College of Engineering, Prince Sultan University, Riyadh 66833, Saudi Arabia; sepaey@hotmail.com or tsebaey@psu.edu.sa; Tel.: +966-(0)-11-494-8644; 2 Mechanical Design and Production Department, Faculty of Engineering, Zagazig University, Zagazig 44519, Egypt

**Keywords:** crushing, thermal exposure, energy absorption, composite tubes

## Abstract

The exposure of polymeric composites to thermal loading is a ubiquitous problem that leads to the degradation of mechanical properties, reducing the service life of an engineered structure, and potentially premature, catastrophic modes of failure. In the current paper, an experimental study is presented in order to investigate the effect of thermal exposure on the crushing performance of carbon fiber-reinforced plastic (CFRP) composite tubes. Specimens of rectangular tubes are subjected to thermal exposure at 90, 120, 150 and 180 °C before being crushed under quasi-static loading. The results showed a reduction in the peak load by increasing the aging temperature up to °C, which is followed by an increase in the peak load at 150 °C, due to post-curing. For the energy absorbed and the specific energy, a sharp reduction is recorded (up to 70% reduction) due to thermal aging. These results showed that the effect of thermal exposure on crashworthiness needs more attention during composites’ design, especially for transportation applications.

## 1. Introduction

Fiber-reinforced plastic (FRP) composites are currently used in several industries, including aerospace, submarine, automotive, construction, and piping, for their high strength and stiffness to weight ratios, chemical stability, and their ability to tailor the strength and stiffness properties by selecting the right fiber orientation of each ply [1,2,3]. The global demand for carbon fiber-reinforced plastic (CFRP) composites over recent decades has increased exponentially, and it yields a four-times increase in CFRP use from 2010 to 2020, as they are being acknowledged as the only current realistic means to coupling high strength and stiffness to low weight [4]. Nevertheless, during the machining, storage, and application, polymeric composite materials are sensitive to climate environment as temperature, oxygen, moisture, and sunlight may affect their aging process. As a result, investigations on their resistance in environmental conditions are essential [5]. Studying the behavior of polymeric composite after exposure to these conditions is crucial for designing the right configuration of material and structure.

Temperature is one of the key factors that affect the behavior of composites. The exposure to temperature results in topography damage with matrix cracks and fiber/matrix interfacial debonding due to chain scission, oxidization, and formation of chromophoric groups of polyamide molecules [6]. All of these physical-chemical changes result in different mechanical behavior, as compared to measurements at ambient conditions. Under tensile loading, Merino-Perez et al. [7] studied CFRP composite plates after exposing it to different aging temperatures (150–350 °C), during 60, 120, and 180 s. The tensile strength increased with the temperature up to the glass transition temperature $T_{g}$, followed by a further decrease at higher temperatures. This increase can be justified to the post-curing effect that incrementally increased the cross-linking density and enhanced the material’s viscoelastic properties. Cao et al. [8] studied the effect of temperature on the tensile properties of different types of FRP, at temperatures from 16 to 200 °C, and concluded that the tensile strengths are dramatically reduced as a function of increasing temperature, up to the $T_{g}$. Liu et al. [9,10] studied the compression properties of unidirectional carbon/epoxy rods and the pyramidal lattice core sandwich panel made of carbon/epoxy at different temperature (−60–260 °C) and the results showed reductions in both the stiffness and strength due to the increase in temperature, but elevated temperatures had a more significant influence on stiffness than cryogenic temperatures. Feng et al. [11] investigated the flexural properties of the glass/epoxy composites at different exposure time (1–90 days) and temperature (45–130 °C). The results showed that the longitudinal flexural modulus increased by 11% after exposure to 90 days. The increased properties were attributed to the post-curing effect of FRP materials. Burks and Kumosa [12] reported the effect of thermal exposure, for up to one year, on the flexural performance, under both static and fatigue loading, of a glass fiber/carbon fiber hybrid composites. It was found that exposure to a temperature near, but below, the $T_{g}$ resulted in a reduction of flexural strength and fatigue performance. From this summary, it can be noted that different loading conditions, temperature, and exposure time resulted in a different material response.

In transportation applications, crashworthiness is the ability of the vehicle structure to protect its occupants during an impact. It is highly dependent on how the materials, construction, and design of the vehicle work together. FRP composites are highly elected for automotive and aerospace structures due to their high specific strength and stiffness, as well as the ability to absorb the impact energy in different failure mechanisms, which improves its crashworthiness [13,14]. Crashworthiness is usually assessed, under compression loading, to the first peak and then to the final densification [15]. Under compression, the interaction between the delaminations and fiber damage can have a considerable detrimental effect on the performance [16]. In addition, in crushing, the newly generated surfaces during the post-crushing stage interfere with the final assessment. Crashworthiness assessment is affected by many aspects, including specimen geometry, fiber orientation, triggering, material, filler material, and test speed [17,18,19,20,21,22,23]. Lau et al. [18] reviewed the effect of the specimen geometry on the crashworthiness and concluded that shape, such as square and circular, showed different energy absorption levels, when tested at the same conditions. In axial crushing, 0.045 ratio of square cross section showed catastrophic crushing, while circular cross section crushed progressively. Mahdi et al. [19] studied the effect of fiber orientation on the quasi-static crushing test and concluded that the 15/−75 is the best in terms of the load-carrying capacity, whereas the 45/−45 is the best for the steady state propagation of the damage. Palanivelu et al. [24] reported that the triggering is important for steady state post-crushing. In addition, different triggering mechanisms result in different energy absorption. Chiu et al. [23] studied the crushing behavior of composite tubes at different strain rates and concluded that the energy absorption is independent of strain rate as the total energy absorption appeared to be largely associated with fiber-dominated fracture, which is independent of strain rate within the studied range. To the best of the author’s knowledge, there is no available study in the literature that assesses the crashworthiness (including the crushing and post-crushing stages) of polymeric-matrix composites after thermal exposure, which is our aim in the current study.

In the current paper, the carbon fiber-reinforced epoxy composite tubes with rectangular cross-section are axially compressed for crashworthiness assessment. The load to the first peak, as well as the energy absorbed and the specific energy, are used in order to check the possible effect of the thermal exposure. Besides, the changes in the failure mechanism due to thermal aging are monitored and compared.

## 2. Specimens and Conditioning

The material used in this study is a commercially available rectangular tube of epoxy-reinforced carbon fiber delivered by Dragon Plate${}^{TM}$ (Elbridge, New York, USA). Two types of carbon fiber are used, as follows: plain weave ($\sigma_{T}$ = 3500 MPa and *E* = 230 GPa) and unidirectional ($\sigma_{T}$ = 4400 MPa and *E* = 235 GPa). For more information about the material and the manufacturing techniques, the manufacturer website can support [25]. The inner and outer layers of the tube are plain weave/epoxy, whereas the middle layers are all zeros to form a stacking sequence of ${\left[\pm 45/0_{3}\right]}_{S}$, with the zero direction points to the axial direction of the tube. Parts from the tubes are cut to measure the fiber volume fraction while using the ignition test [26]. The ignition temperature is 610 °C and the fiber volume fraction ($V_{f}$) of the composite is calculated as:

(1) $$V_{f}=\frac{M_{1}-M_{0}}{\rho_{f}{\dot{V}}_{C}}\times 100$$

where $M_{1}$ is the mass of the container, including the fiber after ignition, $M_{0}$ is the empty container mass, $\rho_{f}$ is the fiber density, and $V_{C}$ is the volume of the composite before ignition. The results of six different samples after ignition showed that the fiber volume fraction of the composite under investigation is 30.5%, with a standard deviation of 1.5%.

The specimens are cut into the desired height of 70 mm while using a computer-controlled abrasive cutting machine. The height of the specimens is selected to optimize the material resources, maintain reasonable crushing stroke, and avoid global buckling of the specimen. A total of 15 test specimens are prepared for the conditioning and, later on, testing. The average dimensions per each test condition and weight are measured and the listed in Table 1, with $T_{E}$ is the exposure temperature, *H* is the height of the samples, *L* and *W* are the cross-sectional dimensions of the rectangle forming the outer profile of the tube, $m_{0}$ and $m_{E}$ are the masses before and after the conditioning, and Δm is the mass losses during the exposure process.

The effect of temperature exposure on crushing behaviors and failure modes of composite tubes is studied using an insulated temperature-controlled air oven to provide high-temperature environments. In real structure applications, composite tubes can be exposed to air at −73 to 80 °C in aerospace, whereas it is exposed to −140 to 120 °C in space applications [27]. In automotive, the temperature around the engine and exhaust can exceed the 200 °C [28]. The temperature range that is used in this analysis is 23 °C (RT) −180 °C, which covers the low temperature to slightly higher than the $T_{g}$ of the material of 166 °C. The exposure time is set constant for the whole analysis to 6 h. As the exposure time is short, the changes in the dimensions can not be recognized, Table 1. Moreover, the differences in the weight of the samples, before and after the exposure, are very small and range between 0.5%–0.6%, which is in agreement with similar studies [5,29] for such a short exposure time.

## 3. Crushing Test Protocol

**Figure 1:**
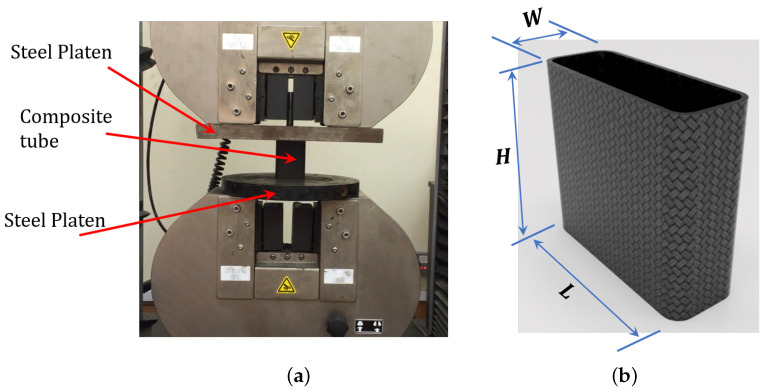
Test setup (**a**) and composite tube details (**b**) of the axial crushing tests.

Quasi-static crushing tests are performed to monitor the failure modes and assess the crashworthiness of the composite tubes after thermal exposure. The tests are conducted using an Instron 8500 digital-testing machine with a full-scale load range of 250 kN. Two steel platens are set parallel to each other before starting the test. Three tests were conducted for each exposure temperature and the average of the three tests was logged. The behavior of each tube under compression is recorded using a camera. The acquisition system of the universal testing machine recorded the load-displacement data at a constant cross head-speed of 5 mm/min., as per the test method found elsewhere [30,31]. Figure 1 shows the test setup and the sample.

**Figure 2:**
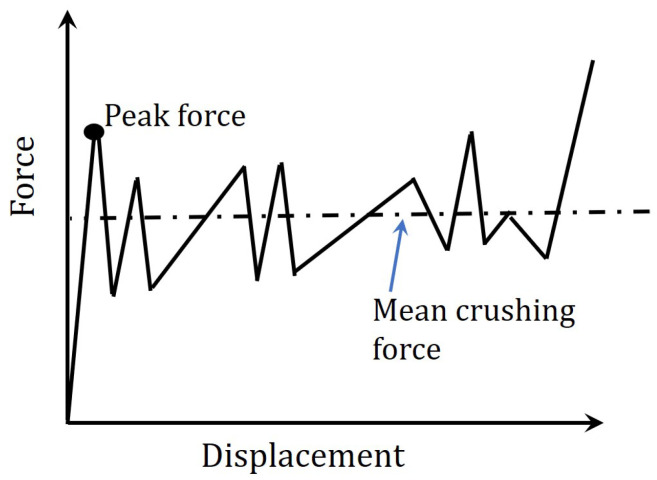
Schematic sketch of the load-displacement profile of composite tube.

The output of the crushing test that is shown in Figure 1 is the load-displacement profile, at which the load usually increases to a certain point and then dropped, as per the first damage/failure. After the first drop, the load experiences some peaks up to the final densification of the sample, as is shown schematically in Figure 2. The parameters used to assess the crashworthiness of the specimen are measured from the load-displacement profile, as:

- Peak force ($P_{i}$) is defined as the load corresponding to the first peak, following the linear portion, in the load-displacement profile.
- Mean crushing force ($P_{m}$) is defined as the average force over a displacement from the peak load $\delta_{Peak}$ to the maximum displacement $\delta_{Max}$ as:
  (2) $$P_{m}=\frac{1}{\delta_{Max}-\delta_{Peak}}\int_{\delta_{Peak}}^{\delta_{Max}}P\;d\delta$$
- Crushing force efficiency (CFE) is defined as the ratio of the mean crushing force ($P_{m}$) to peak crushing force ($P_{i}$), as:
  (3) $$CFE=\frac{P_{m}}{P_{i}}$$
  A high value for crushing force efficiency (CFE) is desirable, because such a structure would minimize the occupants’ injury (if this structure was used as a vehicle crash structure) [32].
- Energy absorption (*E*) is the energy dissipated by the test specimens during the crushing process, up to the maximum displacement and is calculated as:
  (4) $$E=\int_{0}^{\delta_{Max}}P\;d\delta$$
- Specific energy absorption (SEA) is the energy absorbed per unit mass (m), and is calculated as:
  (5) $$SEA=\frac{E}{m}$$
  The higher the value of SEA the higher the efficiency of the material in absorbing energy, during an accident.

## 4. Results and Discussion

**Figure 3:**
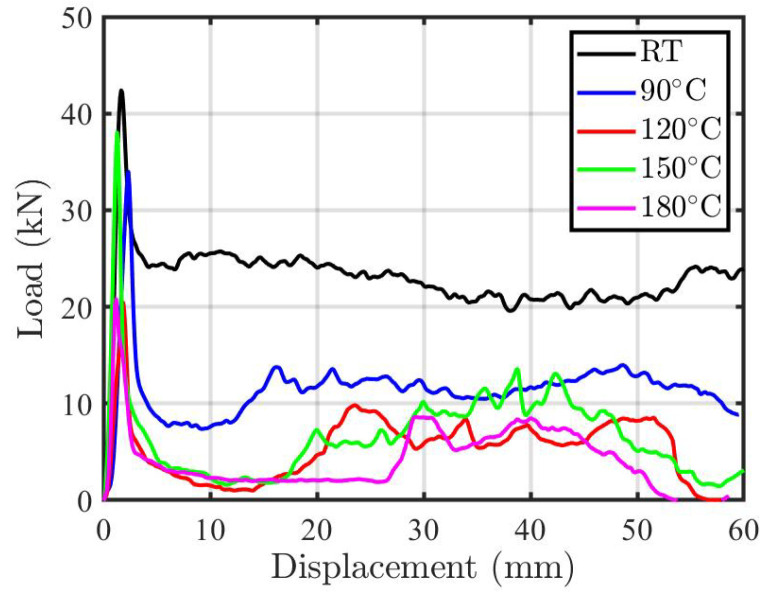
Load-displacement profile of the composite rectangular tubes under crushing after thermal aging.

Figure 3 introduces the load-displacement profile of one sample per each condition. The load-displacement curve profile shows a linear portion at the beginning of the loading that is controlled by the elastic deformation of the tube. This stage (pre-crushing stage) ends at the peak force ($P_{i}$). A considerable drop in the load is recorded after this point, which is the beginning of the second stage (post-crushing stage). During the post crushing stage, most of the well-known damage mechanisms (matrix cracking, fiber-matrix debonding, fiber micro buckling, delaminations, and fiber failure) interact together to form the load-displacement profile [22,32,33]. The end of this stage is called the densification, at which the specimens are not able to carry extra load, and the machine stiffness contributes to the load-carrying capacity. For easy comparing the energy absorption, the data used are limited to 60 mm displacement, just before the densification stage.

The comparison between the results that were obtained for the different aging temperatures shows that the load-carrying capacity is maximum for the specimens without thermal exposure. From the load-displacement plots (Figure 3), it is clear that the load-carrying capacity is reduced by increasing the aging temperature. This is a direct result of the topography damage with matrix cracks and fiber/matrix interfacial debonding due to chain scission, oxidization, and formation of chromophoric groups of polyamide molecules, as it was chemically analyzed by several authors [6,9,10,34]. An exception of this behavior is the high loading capacity of the specimens exposed to 150 °C, at which the load-carrying capacity is high. In the literature similar behavior was reported and justified by the post-curing process that occurs at a temperature close to the $T_{g}$ of the matrix. At this temperature, the consolidation phase occurs, which facilitates the adhesion between the fibers and the matrix [7,11,35,36]. This can justify the improvement in the composite tube’s load-carrying capacity at 150 °C.

**Figure 4:**
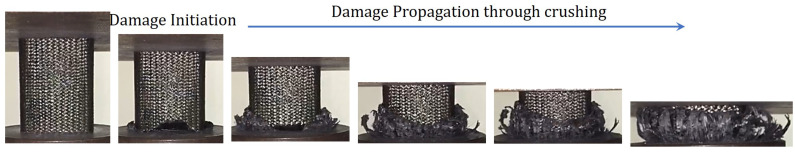
Crushing mode of the CFRP composite tube under different exposure temperature.

**Figure 5:**
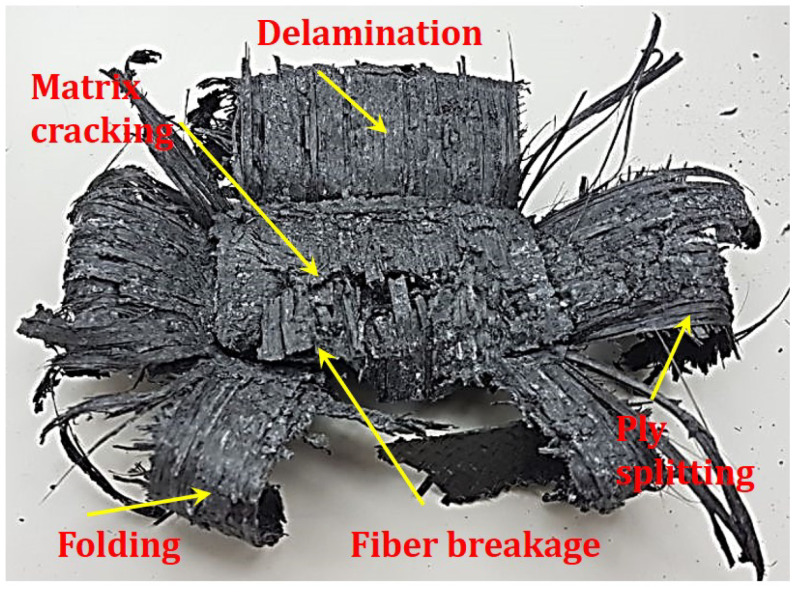
Composite tube after crushing with visible failure mechanisms.

**Figure 6:**
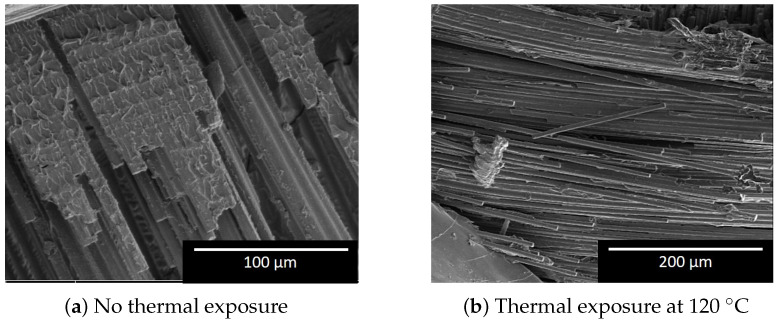
SEM images of the fracture surface of the tubes with and without thermal exposure.

During the tests, the load-displacement profile is reflected in the specimen through different failure mechanisms. At the peak load, the damage initiated on the specimen at the interface with the upper of the lower platen (as this is the weakest point due to specimen cutting in the preparation stage), as in Figure 4. This is followed by the post-crushing stage that is characterized by tearing, fragment, and folding damage at the same side where the damage initiated. It is worth remarking that no significant difference was recorded on the way the specimens behave (in terms of the crushing mode) by changing the exposure temperature. A more detailed view of the specimen after crushing is shown in Figure 5. Besides, SEM microscopy scan images are shown in Figure 6.

The images of the specimen, Figure 5, show most of the known damage mechanism, with the outer $\pm 45^{\circ }$ layer and one 0° layer are folded outward. The rest of the layers are folded inward to fill in the inner hole of the tube. On the scanning electron microscope (SEM) images, Figure 6, the specimens with thermal exposure show a clear fiber surface, which indicates a weak bond between the fiber and the matrix. For specimens without thermal exposure, all the fiber surfaces are covered with matrix, which shows a good bond between the fibers and matrix. Liu et al. [9] and Ma et al. [37] already reported similar behavior.

**Figure 7:**
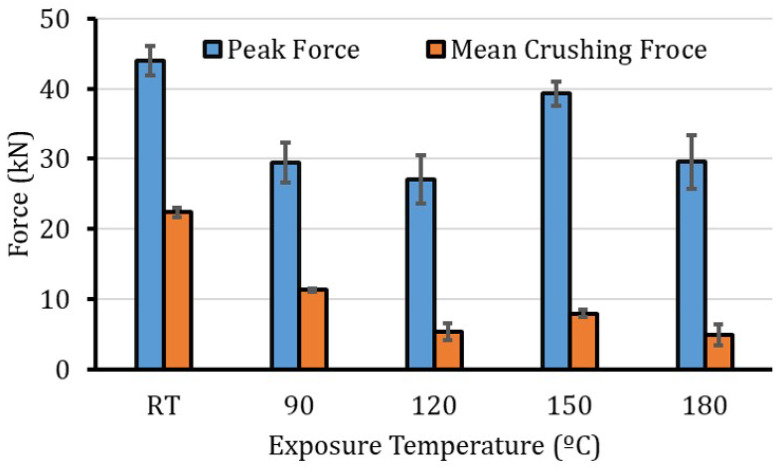
Peak and average crushing force values for the composite specimens at different exposure temperatures.

**Figure 8:**
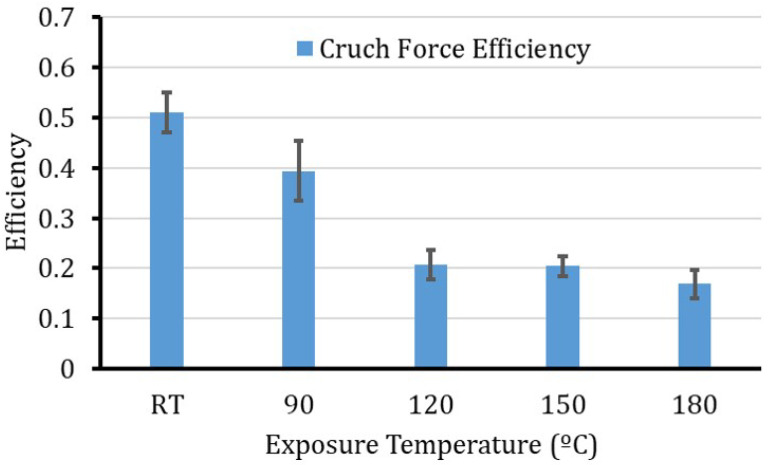
Crush force efficiency of the specimens at different exposure temperatures.

**Figure 9:**
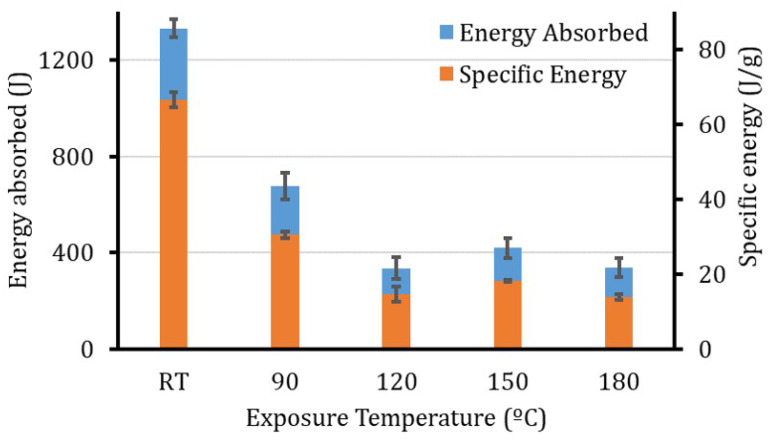
Energy absorbed and specific energy of the specimens after thermal exposure to different temperatures.

Figure 7, Figure 8 and Figure 9 show the crashworthiness assessment parameters. The peak and the average crushing forces are shown in Figure 7. As a reflection of the load-displacement profiles in Figure 3, the peak force decreased as the temperature increased, at the same exposure time. This trend continues until 120 °C and is governed by the matrix and fiber/matrix degradation and mismatch in thermal expansion coefficients. At 150 °C (the closest temperature to the $T_{g}$), the post-curing process [11] increases the crosslinking density and enhanced the properties of the material that yields a high peak and average crushing load. For the 180 °C, the peak and average crushing force reduced as a result of matrix oxidation. The oxidation of epoxy matrix is extensively studied in the literature [27,38,39] and it is associated to the chain scission that is induced by decomposition of hydroperoxides. In addition, it occurs quasi instantaneously, since hydroperoxides formed in epoxy amine networks are unstable due to the vicinity of heteroatoms, which induces the short kinetic chains being an intrinsic characteristic of epoxy. This short summary suggests that the existence of a major or even exclusive chain scission process leads to the ultimate mechanical properties during crushing after high temperature aging.

The Crush force efficiency, Figure 8, shows massive degradation (almost 60%) due to thermal exposure. It is worth remarking that, as a function of the exposure temperature, for the tubes under investigation, most of the degradation occurs from RT to 120 °C. After that, the CFE seems to converge. Even for the 150 °C that has a high $P_{i}$, the ratio between $P_{i}$ and $P_{m}$ does not show huge improvement, as both the Equation (3) parameters (the numerator and denominator) are increasing in similar rates.

For the energy absorbed and the specific energy, Figure 9, the results showed a huge reduction in both of the parameters. Almost 50% of the energy absorption (both *E* and SEA) is lost due to the thermal exposure of the tubes at 90 °C. The degradation continues with a bit of improvement at 150 °C, which is governed by the post-curing processes that can not change the whole trend.

During its service life, CFRP composites can withstand to temperatures up to 300 °C [27,28]. This high temperature for a certain exposure time affects the properties and structure of the material (both physically and chemically), which results in a degradation in the mechanical properties. Under crushing, the damage phenomena are very complicated and governed by the commutative interaction between the different damage mechanisms, which, based on the current analysis, results in a higher effect of the thermal exposure on the energy absorption than that on the tension [8], compression [10], bending [11], and impact [7]. With these results, extra design effort is still needed in composite in order to optimize the tube configuration, not only with the loading conditions, but also with the surrounding environment.

## 5. Conclusions

The current paper pretested an experimental study investigating the effect of thermal exposure on the crashworthiness parameters of rectangular CFRP tubes. The specimens were tested under compression after thermal exposure at 90 °C, 120 °C, 150 °C, and 180 °C, in addition to tests at room temperature. The peak and average crushing forces, the crush force efficiency, the energy absorbed, and the specific energy were used to assess the crashworthiness of the composite tubes.

The results showed a reduction of 40% in the load-carrying capacity (both the peak and the average crushing forces) by increasing the aging temperature from RT to 120 °C. This result can be justified by the lower interface strength associated by thermal aging, as it was proofed by the smooth fracture surface in the SEM images. At 150 °C, the tubes maintained part of its original peak and average crushing load, as a result of the post-curing process. This is followed by another reduction in $P_{i}$ and $P_{m}$ due to matrix oxidation. The crush force efficient lost approximately 60% of its value due to thermal exposure up to 120 °C. After that temperature, the effect of the thermal aging on the CFE is minimal.

For the energy absorbed and the specific energy, the reduction due to thermal aging is more than 70% due to thermal exposure at 120 °C. These reductions are characterized by the matrix cracking and the weak bond between the fibers and the matrix due to thermal exposure. After 120 °C, both the energy absorption and the specific energy slightly increased, as a result of the post-curing process. When considering the huge reduction in the load-carrying capacity and the energy absorption, it is highly recommended to design composite energy absorption devises after considering thermal exposure. This is an environment where all of the structure components are suffering from during their service life.

## Figures and Tables

**Table 1:** Average physical dimensions and masses of the carbon fiber-reinforced plastic (CFRP) composite tubes.

$T_{E}$	*H*	*L*	*W*	$m_{0}$	$m_{E}$	Δm
°C	mm	mm	mm	g	g	g
23	69.7	54.0	28.9	23.51	—	—
90	70.1	53.9	28.6	23.40	23.28	0.12
120	69.7	55.1	29.0	23.00	22.79	0.11
150	70.4	53.9	29.9	23.02	22.85	0.17
180	69.4	28.9	28.9	22.43	22.28	0.15
